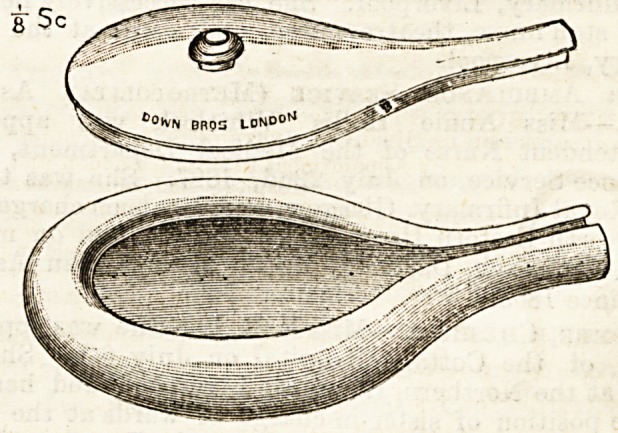# "The Hospital" Nursing Mirror

**Published:** 1897-08-07

**Authors:** 


### "The Hospital" Nursing Mirror.

Being the Nursing Section of "The Hospital."

[Contributions for this Section of "The Hospital" should be addressed to the Editor, The Hospital, 28 & 29, Southampton Street, Strand, London, W.C., and should have the word "Nursing" plainly written in left-hand top corner of the envelope.]

### News from the Nursing World.

### THE NURSING FUND AT ST. GEORGE'S HOSPITAL BOMBAY.

An appeal which must create great sympathy is being made through the Bombay press in aid of the St. George's Hospital Nursing Fund. The plague has proved disastrous to the ordinary income of this fund; patients who would have engaged and paid for the services of the trained nurses fled from the city; up-country sick dared not employ them, so the nursing staff passed through dark days with a failing revenue, which the recent contribution from Government is inadequate to restore. Besides putting the work on a financially sound footing, the committee is desirous of providing a bright, cheerful home for the nurses, where they may rest and recruit their strength between their cases. At present there is only the room below the women's ward for their use—a room condemned as unsuitable for a ward. Then a little money is needed to pension the sick and aged who have spent their lives in the service of the hospital.

### A CHILD'S LIFE.

The action of the North Dublin Guardians with regard to the inquest on the babe Davies is what might have been expected. They are annoyed at the prospect of providing a trained nurse, for the salaries of nurses at the infirmary are £1,000 a year now. They think the temporary medical officer, Dr. Maguire, a faddist to hold that bread is not wholesome in an infant's diet; and would like to know why a child "picked out of the gutter" should need a trained nurse. To these objections it may be pointed out that the difference between the cost of a trained and an untrained nurse is not a heavy item in a bill of £1,000, and that she would most probably save it, healthy children being much cheaper to rear than sickly ones; that Dr. Maguire has, in his opinion of the evils of giving infants farinaceous food, the support of every enlightened physician, nurse, and mother; and, lastly, that, no matter where or when a child be born in the United Kingdom, it is the subject of the Queen, and no man or woman—parent, guardian, or pauper nurse—should be found guilty of neglect or cruelty towards it without answering for their conduct before the tribunals of the nation.

### A RELIGIOUS TEST.

A nice point has recently been discussed by the Town Council of Leicester. The matron of the Borough Fever Hospital dismissed three nurses who were Roman Catholics, whilst in her letter engaging them she had said, "You will be expected to attend Sunday morning service conducted by the hon. chaplain." Unfortunately from these facts it has been inferred that the matron wished to impose a religious test, and dismissed the nurses because of their faith, hence the discussion. The result was summed up humorously by Mr. Wakeley in an "Irish bull." "It has been conclusively proved that, firstly, there has been no religious intolerance at the hospital whatsoever; and, secondly, that directly it was found out they stopped it." It is only fair to the matron to point out that the two nurses do not seem to have raised any objection to her wish until after they had joined the staff.

### GRANTHAM VICTORIA NURSING ASSOCIATION.

Grantham commemorates the Queen's long reign by establishing a nursing association to be called "The Grantham Victoria Nursing Association." The first trustees are the Mayor, Councillor A. Hutchinson, and Mr. W. Hornsby, J.P. Two nurses have been engaged, and a house on St. Peter's Hill is to be ready for them in the autumn.

### ARMY NURSING IN CUBA.

The methods of Spain in waging war are barbarous. Amongst civilised nations the sick and wounded, women, and children are unmolested by the enemy, but this is not so in Cuba. Women and children are regarded as the lawful prey of the Spanish soldiers, who also attack and slaughter the inmates of the Cuban hospitals in cold blood—nurses and patients alike. The atrocities of the "unspeakable" Turk fall short of this, which is only equalled by the Chinese firing, as they did in the late war in the far East, on the surgeons attending the wounded—an action that goaded the Japanese to madness. The Spanish soldiers on the island enjoy the care of the devoted Sisters of the Red Cross, who have their hands full, as sickness is rife amongst them.

### UNCONGENIAL NURSING.

The necessity of raising the standard of infirmary nursing is being forced upon the unwilling ears of guardians by unmistakable evidence both from within and without. Infirmary work is not popular with nurses. We frequently hear of cases like that of Charge Nurse Wylie and Assistant Nurse Dorrance, of the City of London Union Infirmary, who have recently resigned their appointments for the ostensible reason that they "do not like the work"; and papers are occasionally read advising that "the infirmary nurse shall be infirmary trained, as she finds the life dull after the greater stir of the hospital wards." It is incredible that a large proportion of the members of a profession which necessitates the passing of the greater part of their lives by the sick bed, should be dull while there is so much to do; but it is only what might be expected under any system that subjects a trained worker to an incompetent, and in some cases mischievous, rule.

### NURSING AT ST. GEORGE'S HOSPITAL.

In another column will be found an account of a presentation to Mrs. Coster, who for a great number of years has been the matron of St. George's Hospital, Hyde Park Corner. Mrs. Coster belongs to the older school of matrons, of which she has been a worthy and typical example. We have known Mrs. Coster for very many years; have fully appreciated the services she has rendered and the work she has done, and the pathos attaching to much of her life must command the sympathy of all who have any knowledge of it. Mrs. Coster has well earned her pension, and we hope she may live many years to enjoy it in happiness and comfort. With commendable courage, Mr. Timothy Holmes, the treasurer, on the presentation referred to, is reported to have said that the nursing at St. George's Hospital is the best in the world. So much is written about nurses and nursing nowadays that, as the nursing at St. George's Hospital presents many special features which are not to be found at the present day in other London hospitals, we would advise all who are interested in the subject to take an early opportunity of going to St. George's Hospital and studying the system Mr. Holmes referred to. It would be a great gain could we induce our lay confrères, or some of them, to send a well qualified representative to study and compare the nursing system, say, of St. George's Hospital as representing the older type, with the more modern systems which prevail at such institutions as St. Thomas's Hospital, King's College Hospital, and the London Hospital. If such a series of articles were written by a competent person and published in the lay press, they would be calculated to remove many misapprehensions which now clog the wheels of progress.

### READING DESKS IN PUBLIC LIBRARIES.

By the combined action of philanthropists and ratepayers, free libraries have been established in most of the populous centres. These libraries are a great boon, and are highly appreciated by the public. The system of lending books has been brought to something like perfection; the reading-rooms are mostly light and airy, but the reader is obliged to stand at the newspaper desks. For men this is bad enough, but it absolutely precludes women from the advantages offered. To stand for any considerable length of time is injurious to any woman—witness the pale, delicate girls of the shops—but to nurses, and others who must of necessity be much on foot, it is tantalising to have to choose between leaving the news unread, or being exhausted by standing.

### THE CHILDREN'S WING AT DARLINGTON HOSPITAL.

On July 27th Mrs. Edwin Pease formally opened the new children's wing at Darlington Hospital. The wing, which contains twelve beds, is built on to the east end of the hospital, and the cost has been defrayed by the executors of Mr. H. S. W. Cocks, who left £2,500 for this purpose, and an equal sum to be invested as a maintenance fund. The new building is named the "H. A. W. Cocks' Memorial Wing." Very general interest has been excited by the work, and a large number of friends were present at the ceremony.

### THE CHILDREN'S SALON.

The Children's Salon, organised to give children an opportunity to aid in the good work of providing the sick poor with nurses, has resulted in the handing over to the Duke of Westminster the sum of £400.

### THE RESULT OF THE CHELSEA GARDEN FETE.

The Chelsea garden fête was a great success in every way. It has been the means of adding £1,062 to funds of the Homes for Nurses' Association.

### NURSES AT THE ROYAL NATIONAL CONSUMPTION HOSPITAL, VENTNOR.

The nursing of the Royal National Consumption Hospital at Ventnor, of which the foundation-stone of the eleventh block was laid amidst the happiest auspices by H.R.H. Princess Henry of Battenberg, Governor of the Isle of Wight, on Saturday, July 31st, is in the hands of Miss Busby, who was for many years matron of the General Hospital, Birmingham. The staff consists of six charge nurses and eight probationers. The probationers are taken at a slightly younger age than that demanded by the general hospitals, thus affording young people who intend entering the profession an opportunity of learning some of the duties before commencing their more arduous work.

### THE VICTORIA NURSING INSTITUTE, READING.

The preliminary arrangements of the Victoria Nursing Institute, Reading, have been unanimously agreed to by the committee. H.R.H. Princess Christian has expressed her willingness to become patron, and by her advice it has been decided to start the institute on a benevolent basis. It is hoped, however, that the scheme once established, it will be possible to develop the work on provident lines.

### THE WESTMINSTER JUBILEE COMMEMORATION.

The names of trustees who have consented to act for the fund recently collected in Westminster with the object of purchasing a freehold site and erecting a nurses' home are the Duke of Westminster, Sir J. Wolfe Barry, Colonel T. Davies Sewell, Mr. Burdett-Coutts, M.P., and Mr. F. Rose.

### THE FLANNEL SHIRT CLUB.

The first report of the Flannel Shirt Club has recently been issued. The object of its founders is simple, even commonplace; it is to present genuinely necessitous working men who have been ill with a flannel shirt apiece when they leave the hospital. This prosaic charity is of very practical value, and cannot fail to be appreciated by the nurses who have watched their patients' gradual return to health, and who know that, thanks to the timely gift, many dangers to convalescence are avoided. The Countess of Strafford is the president; Miss Lamport, 52, St. John's Wood Road, N.W., is the hon. secretary; Mrs. H. H. Clutton, 2, Portland Place, W., is hon. treasurer; and Messrs. Debenham and Freebody, Wigmore Street, supply flannel for the purpose on advantageous terms. Altogether, it is a nice, useful way of helping the sick in which all may join.

### SHORT ITEMS.

It is reported that Lady Henry Somerset has resigned her position as president of the British Women's Temperance Association, in consequence of her holding different views to her associates on the subject of the Cantonments Act.—The site given by Sir Henry Watson for the Worksop Cottage Hospital seems generally approved, and the building fund creeps steadily up, but more money is still needed. —The sisters on the nursing staff of the Rangoon Hosp'tal are much disappointed at not being invited to the public ball at Government House, as has hitherto be n the ann-al cu'tom. It is a pity that the omission should have occurred in this year of national rejoicings.

### Practical Aspects of a Nurse's Life.

By Sister Grace.

### PROBATIONERS IN MEDICAL WARDS—

(*continued*).

### IV.—Pneumonia.

You will not be long in medical work before you meet with pneumonia and typhoid fever. The former is, as you know, inflammation of the lungs—it may be on one or both sides. If on one side only, you will find that the patient usually lies on that side, so as to give the unaffected lung more freedom of action; but if both sides are touched he will prefer to lie on his back with the shoulders raised. The expectoration is characteristic—jelly-like in substance, very tenacious, and rust-coloured. In some fatal cases there is no expectoration, the patient being unable to cough up anything. The doctor will need frequently to ascertain the state of the lungs by percussion, and a clean towel will be wanted to dry the patient's back and chest. Do not forget these trifles. Pay attention the first time you are told anything fresh, and never require the instruction to be repeated, and in waiting upon the sister, try, by observation and the use of your common sense, to anticipate her wishes; at the same time do not commit the error (in your anxiety to be helpful and quick) of only partially listening to what she says before rushing off in headlong zeal to carry out orders you have only half grasped; this kind of nurse, zealous but without discretion, is a trial to all. When percussion is going on stand motionless and don't fidget from one foot to the other. It is surprising the want of observation and common sense displayed by some nurses in persistently, and one would almost think of malice aforethought, choosing the moment when a doctor is sounding a chest, to creep across the room in noisy shoes, or settle receivers in the rack, or turn a tap in an adjacent scullery.

To return to pneumonia, the treatment by jacket poultices is not so general as it was, though still often resorted to, and practice alone can teach perfection in their making and application. I was taught by a sister who had brought the making of jacket poultices to such perfection as to be really among the fine arts, and for that and much useful instruction as to the nursing of typhoid fever, in which she was singularly successful, I shall always be grateful. Pneumonia yields early to treatment or speedily terminates fatally; it does not, therefore, tax the patience and perseverance of a nurse in the same way as typhoid fever.

### Typhoid Fever is, by all who understand nursing it, considered to be what Mrs. Gamp called "the fine work" of medical nursing. One reason of this undoubtedly is that the recovery of the patient largely depends on the skill, perseverance, and untiring devotion of the nurse. All doctors are ready to admit this, and anxious to secure for the sufferer the most skilled attendance possible. You will have learnt some simple facts concerning this disease, such as that the infection is carried in the secretions, therefore you will know that great care is required in keeping all utensils used apart and carefully disinfected. Having been once told of the necessity for care in these matters, it must be left to your own sense of duty and obedience to carry out your instructions. If you are not conscientious in these things, you will endanger your own life and the lives of others. Of the appearance and symptoms of enteric or typhoid fever you have at present nothing to do, but it may interest you to know that if there is a persistent evening temperature of from 102 to 104, attended by more or less diarrhœa, the motions presenting a yeasty appearance, it is probable that the fever may declare itself ere long by the advent of a few small round red spots, separate from each other, slightly raised, and temporarily disappearing on pressure by the finger. The disease seriously affects the intestines, and therefore the patient is kept on fluid diet for some time, which period varies with his condition and the particular views of the doctor in attendance. Milk is the staple food generally, mixed with barley water. During the worst stage it is often difficult to persuade the sufferer to take sufficient nourishment, and if he does not do so exhaustion speedily ends the case. You must try every means to get down the requisite amount. An adult should take from 2½ to 4 pints in the 24 hours. As he begins to improve your difficulties will lie in the opposite direction; he will want unlimited milk, and soon be ravenously hungry. In hospitals this constitutes a danger, for fellow patients pity his apparently starved condition, and unless closely watched are likely to give him forbidden morsels, and on visiting days it is a serious anxiety. Many a case has relapsed and ended fatally, without apparently adequate cause, until it is discovered that friends have smuggled in food. As a probationer I remember going into a side ward and finding a typhoid patient in the act of swallowing a large mouthful of a pallid, unwholesome looking jam tart. The sister had left him in my charge, and I had never left him during the visiting hour; nevertheless the friends had baffled me and insinuated this delicacy into his bed whilst tenderly holding his hand. My suspicions being aroused, I made a thorough search of the bed, and was rewarded by finding half a pound of dried figs and three plugs of tobacco to chew in his shirt. Those suffering from typhoid fever must maintain a recumbent position; the ulcerated state of the intestines makes it fatal to put any strain upon them, such as is caused by sitting up; perforation would probably take place, and death ensue in a very short time. Many doctors order tepid sponging whenever the temperature rises above 103, which often gives relief and induces refreshing sleep. You will be required to assist the sister in doing this, and so learn how to manage it with skill and celerity.

### Presentation to Mrs. Coster.

On Tuesday evening, July 27th, the past and present members of the nursing staff of St. George's Hospital assembled in the board-room for the purpose of presenting Mrs. Coster with a very handsome gift of plate on her resignation of office. Mr. Timothy Holmes, treasurer of the hospital, made the presentation on behalf of the nurses, and in dignified and impressive terms alluded to the value of Mrs. Coster's services to the hospital During the long period of years she had held office the nursing had steadily improved till now, in his opinion, it was the best in the world. English nursing was the best, and St. George's Hospital stood in respect of its nursing in the front rank. The beautiful gift which he had the pleasure of offering for Mrs. Coster's acceptance had been subscribed to by nurses in all quarters of the globe who had had the privilege of being trained by her. The gift was an honourable one to both donor and recipient, and he trusted Mrs. Coster would long live to enjoy and use it. Mrs. Coster, in reply, said: I thank my nurses from the bottom of my heart for their beautiful present to me. Words cannot express my deep gratitude, but I feel it none the less deeply. Whatever work I may have done in the hospital, it could not have been accomplished without the loyal co-operation of the nurses, and in thanking you all once more for your beautiful present, I trust that you will often come and help me to use it. The gift, which included a handsome silver coffee pot and complete set of plate for the table from the nursing staff, and a dainty white china tea and coffee service from the servants and ward helpers, was then duly inspected and admired by those present. A very pleasant and sociable half-hour followed, after which the company gradually dispersed.

### Post-Graduate Clinics for Nurses.

BY A TRAINED NURSE.

### XXV.—A CONVALESCENT REST CASE

By this time the patient was distinctly better. As a rule, after five or six days of the treatment a wonderful change comes over the temper and the spirit of the patient. From jerky, restless persons—themselves object lessons of the possibility of perpetual motion, they begin to develop a wholesome idleness. They will lie for an hour or two like logs—the tidy condition of their bedclothes being in a sense the thermometer which registers their restfulness or the reverse. Gradually they cease from their weary habit of asking questions. And the desire to discuss their symptoms and emotions by the hour shows a gradual improvement. The masseuse was directed—when the bran and castor oil were discontinued—to direct special attention to abdominal and liver massage. For this in the United States is relied on for curing habitual constipation far more than is a resort to chronic aperients. Mrs. V., in addition to indulging largely in aperients, had been long accustomed to an almost daily use of the enema. And it was invariably found that the constipation produced eventually from constant enemata was far worse than that which resulted from an habitual indulgence in aperient medicine. Now that the state of her nerves was tranquilised, it was adjudged that she might be allowed to have a slight current of electricity. It was thought the stimulus afforded by the current might have some effect in counteracting the constipation which so materially kept her from benefiting as much as she should by her treatment. As Dr. Weir-Mitchell says, "When we put patients in bed and forbid them to use or make use of their muscles, we at once lessen appetite, weaken digestion in many cases, set up constipation, and enfeeble circulation." Of course, these are the reasons underlying the necessity for massage in rest cases—that method whereby passive exercise is obtained without fatigue to the individual whose muscles are moved for him. In the majority of cases massage more than makes up for the loss of exercise involved in rest-cure, and most patients are astonished to find they are less constipated in bed than they were when up and about and taking exercise.

But Mrs. V. proved an exception to this rule, and it was found necessary to give her every night an enema of 10 oz. of linseed tea. The want of tone in the bowel may be best illustrated by the fact that the patient frequently retained this injection the whole night, and relief was obtained in the morning only by the further injection of a quart of hot water. To vary the linseed tea, and for the sake of its more astringent quality, ox-gall was, after a few days, substituted, and the patient was instructed to, and never seemed to find the least difficulty in, retaining this during the night. It was hoped the local effect of the ox-gall in contact so long with the bowel would have a tonic effect.

I much regret that a small rent in the page of the book in which the notes of this case are set forth prevents me from being able to decipher the amount of ox-gall which was prescribed for the enema, and as it was the only instance I met with of the use of such a remedy, I am afraid to trust to my memory for the dose.

Mrs. V. had gained considerably in weight, but this was a circumstance only, for her treatment was not directed towards flesh-making, since she was a rather heavy, muscular woman. "Rest-cure" in her case had been for the purpose of restoring a healthy mental balance, to enable her to regain a self-control she had never possessed in a high degree, to overcome a confirmed sleeplessness, and to remedy a long-standing constipation. The result showed great improvement in the three first conditions, but the serious constipation was only mildly mitigated—never entirely removed. At this time the patient was taking 70 oz. of peptonised milk in the 24 hours. This amount of pre-digested milk was, roughly speaking, held quite equivalent to three quarts or more of fresh milk, and as she often complained of hunger, she was allowed a thin piece of bread and butter three times daily, and gradually by increasing the amount of solid food taken and lessening the milk by about 5 oz. daily, she was brought back to house diet and one quart of peptonised milk each day.

Abdominal and liver massage, coupled with careful dieting and rest, having failed to relieve to any appreciable extent the constipation so troublesome a complication in Mrs. V.'s convalescence, resort was had to electrical treatment. Galvanism was applied daily for ten minutes to the rectum. As was usual with every fresh remedy, this proved effectual for a few days, and then the patient reverted to the original condition of constipation.

She rather liked the flavour of peptonised milk, which she said "hadn't quite the milky flavour of milk." But the doctors were unwilling to allow her to continue on peptonised milk, and this for two reasons, the first reason being that indulgence for too long in pre-digested food has a very weakening effect on the digestive organs. And again, the amount of soda used in the peptonising process is apt to have a depressant effect when taken over a long period. In order to vary the "milk taste," which proves so monotonous, we made it an invariable rule, and generally before our patients had time to develop the customary feeling of nausea at the sight of the "eternal glass of milk" to flavour this in different ways, so as to keep up the appetite and give the patients the charm and benefit of change. The addition of barley water gives quite a different taste, and lime water, again, makes a change, and both of these, of course, cause the milk to be easier of digestion. Infinitesimal quantities of tea or coffee used alternately or occasionally turn milk into quite a new beverage, while flavouring with caramel, or a very tiny proportion, about a salt-spoon of Liebig's extract of meat to each tumbler makes very pleasant variety, as does also the addition of one of the many aerated waters whose flavours differ.

It was eight weeks before Mrs. V. began her convalescence by sitting up for ten minutes the first day and increasing ten minutes every day. And at the end of ten weeks she was discharged, a very different woman from when she was admitted. She had gained much in flesh and weight; she was entirely cured of insomnia, "jerking," and constant restlessness and irritability. Her habit of constipation was relieved but not cured, and her serious digestive disturbances were much alleviated. By taking care in the avoidance of indigestible compounds she was able now to take fairly average meals with good appetite, without after-pain or flatulence. But she was advised always to lie down for fifteen minutes before a full meal and for half an hour afterwards.

And so Mrs. V—— was restored to her friends—very much improved in health and condition. But she was not one of our "triumphs"—and it is doubtful whether she could ever be entirely cured even under the best conditions. For her doctors were convinced that her capacity for mental worry and her nervous excitability were too deep-rooted to admit of change.

But such as the cure was we all had done our best for one of the most trying patients I ever met with.

### "THE HOSPITAL" CONVALESCENT FUND.

We thank Nurse K. Tilt for her kind contribution of 3s. to "The Hospital" Convalescent Fund.

### Everybody's Opinion.

[Correspondence on all subjects is invited, but we cannot in any way be responsible for the opinions expressed by our correspondents. No communication can be entertained if the name and address of the correspondent is not given, or unless one side of the paper only is written on.]

### BADGES.

"A Nurse" writes: I think it a very good suggestion about the badges. I am sure all trained nurses would only be too glad to pay the required fee. May I suggest also that it would be nice if each nurse could have her name and where she was trained engraved on the silver badge to be worn outside the uniform.

### FALSE TESTIMONIALS.

"A Sufferer" writes: I think it is time that there should be some steps taken to prevent matrons and others giving false references to nurses who have that terrible and disgraceful failing of taking stimulants to excess. They may be good nurses, and, as such, people speak of them, but omit to mention their failing, thereby causing the general public and many homes to suffer for their misdeeds—for intemperance and other faults of character go hand-in-hand. For example, a nurse joins a home (possibly holding a good certificate of training, and has obtained from some matron a reference which speaks of everything but the one fault, most likely with the view of not wishing to injure the nurse. The first case the nurse goes to all will be well; but the next she enters a house where such an idea as a nurse not being sober would have been unheard of. Here she gives way to her craving, disgraces herself, and is sent back to the home, thereby losing caste for the home with both patients and doctors. Why should the innocent suffer? Something should be done.

### "SIR" OR "DOCTOR."

"A Hospital Surgeon" writes: In addressing this letter to you, I do so in the hope that no persons but nurses will read it. I am a hospital surgeon; I am not an M.D., nor do I desire that honourable distinction; but when I now go to an operation at a private house, or even in a surgical home, the nurse, in response to remarks from me, replies, "Yes, doctor"; "No, doctor." What is the reason of this? It never used to be so. When I was a house surgeon I always addressed my chief as "Sir." There is nothing derogatory in the use of this little word. The lieutenant when on duty calls his captain "Sir," and thus also does the captain address his colonel. A ward sister of the social position of a lady—I mean a lady by birth and education—calls the surgeon of her ward "Sir"; yet it seems now to be the fashion of the private nurse to ignore that word altogether, and I, personally, regard this custom as the offspring either of ignorance or bad manners.

### HOMES FOR AGED NURSES.

"Nurse E." writes: Will you again kindly allow me space for a few lines in your valuable paper? I am so grieved that the scheme for providing a home for nurses seems to be falling through. I think the Pension Fund a most excellent thing, and would strongly recommend all young nurses to join it, and only regret that I did not do so myself. It was only started after I left London to take up private nursing here, and not seeing any nursing paper until the last two or three years it did not come under my notice at all until it was too late. Now, verging on 50, and being incapacitated for work through ill-health for the last two and a-half years, I have experienced the loneliness and discomfort of lodgings with very limited means. I had saved a little, or I could not have gone on so far, but there is a future to face when the savings are gone, and I can sympathise with those poor nurses who through age or ill-health are unable to work, and who are without homes or means. Truly, their case is sad! Unfortunately, though so good for the present day nurses, the Pension Fund does not help nurses of 50 or 60 when it is a case of present, not future need. I wish so much that a home, like the one at St. Leonards (Albert House), for ladies of limited means, which provides two furnished rooms, with cooking and attendance, for 8s. a week, could be opened for nurses in Bournemouth. Of course, this sum could not include board, but I daresay there are many who, like myself, could afford, instead of that sum, say, 7s. a week and then board themselves, and would be glad to do so. They would each be free, and yet have the companionship of other nurses, who would be kind and neighbourly. The friends to whom I have mentioned the idea are of opinion that if such a home were once started it would be successful and in a time almost self-supporting.

### A PROTRACTED EXISTENCE.

"Queen's Nurse" writes: A district nurse would be glad to know if, in the experience of other nurses, the following case of protracted existence, almost without taking nourishment, is not of an exceptional nature: A poor woman, aged 65, was suffering from a vaginal malignant growth. She was admitted into a local hospital, and, after several weeks, refusing to undergo a critical operation, was discharged as incurable. About a month after her return home she rapidly became worse, the pain became constant and intense, and she developed "thrush," being then to all appearance, and in the opinion of the doctor in attendance, in a dying condition. Simultaneously with the "thrush" a dark coloured raised rash covered her body. For three days the throat appeared closed, and she could not take any nourishment at all. The tongue and throat were painted with the usual remedies, and on the fourth day an improved condition appeared and the patient was able to take some nourishing drinks. She was partially comatose, but rallied and could reply when she was addressed until the last week. She had frequent paroxysms of severe pain, followed by collapse, and was not kept under the influence of any sedative. After taking nourishment for a few days she ceased to take anything more than a spoonful of weak brandy and water, or more often of water only occasionally, and, in that condition, she lived for a little over six weeks. The district nurse during this time visited daily. The patient showed very little decline of bodily strength, and, if left alone, would get out of bed and sit up. She continued to do this until within four days of her death. There was a continuous offensive vaginal discharge until within the last week, when it entirely ceased.

*** The question is an interesting and very proper one. It is extraordinary how long people are found in some cases to live without food if only they can take water and do not suffer much from pain or fever. In this case, however, there seems to have been pain. In some cases of protracted existence, such as the one described above, nutritive enemata have been given and have been thought to have kept the patients alive. The opinion, however, is strongly held by some that these enemata chiefly act by supplying fluid. We should be glad to hear of well-authenticated cases of prolonged existence without food which have come under the personal observation of nurses.—Ed. *T. H.*

### Novelties for Nurses.

### NURSE BRIERLEY'S PATENT CLEANSABLE BED-PAN.

The above illustration shows an improved form of bed-pan, made at the suggestion of a practical nurse. The difficulty attending the cleansing of the ordinary bed-pan is well known by those in the habit of using them, but is effectively overcome in the present model by an opening along the entire length of the handle portion. The new pan is also a little deeper in the centre than those in common use, but, being gradually sloped from the sides, is equally convenient for placing under the patient. The invention has been patented, and is manufactured by Down Bros., 21, St. Thomas's Street, London, S.E.

THE "VERACITY" WATCH.

Mr. J. N. Masters, Hope House, Rye, Sussex, has drawn our attention to his hospital watches, designed especially for the use of doctors, matrons, and nurses. These watches are of hall-marked gold or silver and have centre-seconds hands for timing the pulse. The "nurse's watch" is of convenient size and nice appearance; it costs in silver from £2 2s., and in gold from £6 6s., with keyless winding; and from 30s. silver and £5 5s. gold with key winding arrangements. The doctor's watch is much the same, but of a larger size; it also possesses what is known as a "compensation" balance by which the watch is rendered invariable by changes of temperature; the price is £6 6s. silver, and £16 16s. gold. The title of "Veracity" is registered by this maker for his watches. He claims that it is a true one because his watches keep such excellent time.

### Presentations.

Iver, Langley, and Denham Cottage Hospital, Bucks.—On Monday, July 26th, the ladies of the committee of the Iver, Langley, and Denham Cottage Hospital, Bucks, presented Nurse Elsby with a silver Jubilee medal, accompanied with the following note: "This Jubilee medal is presented to Nurse Elsby in remembrance of the sixtieth year of the glorious reign of Her Gracious Majesty Queen Victoria, and also as a memento of Nurse Elsby's ten years' faithful and valuable service to the Iver, Langley, and Denham Cottage Hospital, by the Dowager Lady Churchill, the Lady Sybil Montgomerie, Mrs. Way, and Mrs. Lloyd."

Miss S. A. Warburton, who is resigning her position as Matron of the City of London Infirmary to take the post of Lady Superintendent of the North Staffordshire Infirmary, Stoke-upon-Trent, has been presented by her nursing staff, as a token of their good-will and esteem, with a handsome silver cake basket. In Miss Warburton the City of London loses the services of a devoted and energetic worker, and the nurses a kind and sympathetic friend.

Bishop Auckland.—Nurse Violet Wood has been presented with a handsome umbrella by Mr. Joseph Lingford, J.P., on behalf of the St. John Ambulance Association, as an acknowledgment for two lectures and demonstrations to the men.

### Appointments.

MATRONS.

Wigan Infirmary.—Miss Georgina H. Sked was appointed Assistant Matron of the Royal Albert Edward Infirmary in July, 1897. She was trained for one year at the Royal Hospital, Edinburgh, and for four years at the Royal Infirmary, Liverpool. She has successively held the posts of staff nurse, theatre nurse, and sister at the Royal Infirmary, Liverpool.

River Ambulance Service (Metropolitan Asylums Board).—Miss Annie Hollis Whitlock was appointed Superintendent Nurse of the Medical Department, River Ambulance Service, on July 22nd, 1897. She was trained at the Royal Infirmary, Glasgow, and has been charge nurse at the North-Eastern Hospital, Tottenham, and on medical ships, Lady Reach, Dartford, under Metropolitan Asylums Board since 1895.

Seacombe, Cheshire.—Miss E. B. I. Bailie was appointed Matron of the Cottage Hospital on July 6th. She was trained at the Northern Hospital, Liverpool, and has since held the position of sister-in-charge of wards at the Blackburn Infirmary, and also at the South Devon and East Cornwall Hospital, Plymouth.

Dufftown.—Miss Elizabeth Law was appointed Matron of the Stephen Cottage Hospital on August 2nd. She was trained at the Royal Aberdeen Hospital for Sick Children, and has since been nurse in the same institution.

### Minor Appointments.

Isolation Hospital, Blaby.—Miss Sara Baker has been appointed Charge Nurse of the above hospital. Miss Baker was trained in the Bristol General Hospital for some years, and afterwards was on the staff of the Royal Derbyshire Nursing Institution.

Herne Bay.—Miss Annie Robertson was appointed Charge Nurse of the Convalescent Home (S. M. School District) on July 22nd. She was trained at the Royal Hospital for Children, Aberdeen, for three years, and was subsequently charge nurse in the same institution.

Infirmary and Dispensary, Bolton.—Miss A. E. Brown was appointed on July 29th Sister to the above infirmary. She was trained at Radcliffe Infirmary, Oxford, and has held the post of sister at the Derbyshire Royal Infirmary.

Dearnley, Rochdale.—Miss Emma Elaine Evans was appointed Superintendent Nurse of the workhouse infirmary on July 8th, 1897. She was trained at St. Mary's Hospital, Manchester.

Croydon.—Miss May Isabel Wildsmith was appointed Night Charge Nurse of the epileptic ward of Croydon Union Infirmary.

### Notes and Queries.

*What Constitutes a Hospital?*

(329) Will you kindly tell me what constitutes a hospital? (2) Also a recognised hospital? (3) Is a registrar of births and deaths supposed to attend gratis any maternity home or hospital where there are over fifty births per year.—*H.B.*

(1) It is impossible to say what constitutes a hospital, as the definition has not yet been specified. (2) Recognised by whom? (3) By courtesy only.

*Abdominal Specialists.*

(330) I should be much obliged if you could let me know the address of one or two good doctors skilled in abdominal cases whose fees are not very high.—*A Country Nurse.*

Consult your own medical man, or the list of medical men in attendance at one of the special hospitals for the class of disease you mention.

*Training in a Children's Hospital.*

(331) Could you kindly inform me of any children's hospital or training home in or near London where young probationers are taken? I am just 20.—*C. F. B.*

Consult Honnor Morten's "How to Become a Nurse," published by the Scientific Press, 28 & 29, Southampton Street, Strand. It contains full information upon the subject. The Matron, Royal National Hospital for diseases of the Chest, Ventnor, takes probationers of your age.

*Hospital for Consumption.*

(332) Can you kindly tell me of any hospital in Cornwall or Devon suitable for youth in early stage of consumption, free, or on payment of small sum?—*Nurse Green.*

The Western Hospital for Patients of Consumptive Tendency, Torquay, Devonshire, has 40 beds, and admits patients over 15 years of age with subscriber's letter at 5s. per week, and without letter for 10s. a week. The Royal National Hospital for Diseases of the Chest, Ventnor, Isle of Wight, takes patients from all parts of the United Kingdom; a subscriber's letter is necessary, and the patient pays 10s. a week.

*The Climate of Wiesbaden.*

(333) Can you give me any information about the climate of Wiesbaden, whether bracing or not? (2) Also the names of English doctors there. I want a bracing climate at one of the baths for rheumatoid arthritis.—*Lilla Hotson.*

Wiesbaden at this time of year is far from possessing a bracing climate. (2) You would possibly find the information you want in the "Dictionary of Mineral Waters and Climatic Health Resorts, Sea Baths, and Hydropathic Establishments," recently published by Messrs. Kegan Paul, Trench, and Co., price 2s. 6d.

*A Probationer's Duty.*

(334) Would you kindly inform me whether I have authority to order the probationer to give a bath to a sick man after ten o'clock at night where there is neither a male nurse nor a porter? And have the guardians power to call upon me to resign for doing so, after having been in their service between five and six years?—*One in Trouble.*

The propriety of the order depends on the age and experience of the assistant nurse. When ordered she must obey, but the guardians are quite within their rights in dismissing a nurse for what they may consider an act of impropriety.

*The Newest Dictionary.*

If Sister Gertrude will repeat her query and write it distinctly, we shall be pleased to make further inquiries, and let her know the result.

## Figures and Tables

**Figure f1:**